# Public Appreciation of the Dental Profession

**Published:** 1905-02-15

**Authors:** J. E. Nyman


### PUBLIC APPRECIATION OF THE DENTAL PROFESSION.

BY J. E. NYMAN, D.D.S.

The standing of the profession before the public depends entirely on how we bear ourselves while we are in the public eye. I think that the profession on the whole does lack what we may call *esprit de corps*. We do not give evidence of learning and culture, which is always appreciated, nor do we give evidence of being men of experience, things of great value in determining what shall be our relative standing in the eyes of the public at large. The public will accord us a much higher rating if we give evidence of learning, culture and experience. This is not better demonstrated than by the artist Whisler, of London, who brought suit against a man to collect a bill of $1,000 for one of his paintings. The man insisted upon a jury, feeling, of course, that a public man would readily recognize the injustice of such a charge for so little time spent. This charge seemed to the man a ridiculous one. Finally Whisler himself was put upon the stand, his attorney asked him a few questions and turned him over to the defendant for cross-examination, and the defendant's attorney said:

"How long did it take you to paint that picture?"

Mr. Whisler answered, "A part of one morning, sir."

"And you have the audacity to ask $1,000 of this man for a part of one morning's work?"

"No, sir; but I have asked him $1,000 for the experience that is based on the work of twenty years."

And in spite of the fact that the defendant's attorney appealed to the jury in every way he could, the jury rendered the unanimous verdict for $1,000. His own bearing and the reply he made gave evidence of the fact that his ability to paint the picture was due to the fact that he had worked twenty years. He gave evidence of learning, culture and experience, both in his bearing and in his reply. And, gentlemen, I feel that the public at large would accord us a much higher rating—would see greater value in our services—if, in our conduct before them, we give evidence of these three things. Now, then, we all know that a man cannot combine a high professional standing with drone business principles, but *vice versa.* For instance, if a man works at his life-work with the predominating idea that he will come as near perfection in all that he does as is possible, no man will criticise him. If he is an artist instead of an artisan, people will accord him the highest possible value for what he does, for in the history of the race it is the artist that has always given all that is best for the welfare of mankind and never the artisan. It has been the man who has looked to the perfection of things, who has accomplished for man all that can be done for his welfare. Then let our conduct before the public, as a profession, be such as to command their respect, and not their disregard. We should avoid selfish displays and displays of egotism. The truly scientific man cares not for sensational displays, no matter how it can be justified. He never gives any evidence of the fact that he himself really thinks he is great. If you will notice, egotistical displays are always the best means of showing the incompentency of a man. A man of learning should avoid these kind of displays.

A man who invents is in a certain sense a discoverer, and discovery always carries with it certain rights of possession. It matters not in what field the discovery is made, whether it be in the field of learning or commerce. I think it is perfectly just that an inventor should have the right of his possessions. I think it would be a far greater wrong to deny a man these rights which are his. There are, however, two kinds of patents—patents on appliances and patents on processes. I do not believe in the patenting of a mere knowledge of what to do and how to do it by professional men, where really nothing is essential but a study of principles, but when a man really discovers anything, he should be entitled to the benefit of it. Inventing a dental instrument is seldom the result of accidental discovery, or of a mere dream, but means the result of hours and hours of labor, and if this labor were devoted to something else—something in the way of professional work—the man would have certain pecuniary returns. Therefore, he should also have returns from the invention of an instrument. It is almost the same in the medical profession as it is in ours. I know men of wide reputation who hold patents on instruments. They have taken out these patents, not only for pecuniary reward, but to protect the profession from spurious articles, and if it were not for the patent, there would be all kinds of articles made. By having these patents a man who infringes on these rights can be punished.

I believe that there are too many dental journals. If we could have one good journal published each week, we could have the valuable transactions of all societies in the country published, and still have plenty of room for contributed articles, as well as articles on research work. Then if our profession supported that publication and no other, we would soon have a stop put to the practice which is now so much in vogue, of every supply house that comes into existence starting up a dental publication immediately, and hiring some man to run it. These dental journals which are put out by these supply houses do not improve our standing, nor dignify the profession in the eyes of other professions, or in the eyes of the people.

I feel that much good could be done in raising the standard of the profession, if, in our colleges, some definite time was set apart for the study of ethics and professional standing, and I would have the most entertaining man in the faculty deliver lectures on these subjects, one who could absolutely convince his hearers of the justice and value of what he said. We should take as high ethical standing as the medical profession. In fact, I feel that our profession is far more scientifically backed than the medical profession. and I believe that we truly relieve suffering, even more than the medical profession, and we give certain definite permanent results that do not come with the medical or legal profession. We do not stand before the public in exactly the same way and on the same high plane as is awarded to the medical and legal profession. And why? The public at large feels that the lawyer stands for their liberty and life and possibly their earthly possessions. He knows it means the loss of one of these things if he gets into trouble; and the public at large regards the physician and surgeon as the man who stand between life and death, or between him and death, and so, of course, they award him a higher rating than they award the dentist. In our profession the loss of teeth can be replaced by temporary ones and, although not as good as their own teeth, it still does not mean life and death to them. This is the main reason why the public at large hold the doctor and lawyer in such high esteem and put the dentist on such an inferior rating. There is no reason why we should not lift ourselves up to the highest possible plane and receive the highest possible rating in the eyes of the public, so to speak, and we can do this by maintaining an independent spirit and giving evidence of learning, culture and ability.—*Summary.*

